# MicroRNA-155 contributes to host immunity against *Toxoplasma gondii*

**DOI:** 10.1051/parasite/2021082

**Published:** 2021-12-15

**Authors:** Yanan Xu, Junhua Wu, Xiaoqi Yuan, Wenyuan Liu, Jiewen Pan, Binbin Xu

**Affiliations:** Ningbo Women and Children’s Hospital Ningbo Zhejiang PR China

**Keywords:** *Toxoplasma gondii*, Toxoplasmosis, miR-155

## Abstract

*Toxoplasma gondii* is well known to infect almost all avian and mammalian species including humans, with worldwide distribution. This protozoan parasite can cause serious toxoplasmosis, posing with a risk to public health. The role of microRNAs in the pathogenesis of *T. gondii* has not been well described. The aim of the present study was to investigate the role of microRNA-155 (miR-155) in mediating innate and adaptive immune responses during *T. gondii* infection in mice models. The survival and parasite burden in *T. gondii*-infected miR-155^−/−^ and wild-type (WT) C57BL6 mice were compared. In these two mouse models, ELISA tests were used for analysis of Th1-associated, Th2-associated, and Th17-associated cytokines, and flow cytometry was used for analysis of the subpopulations of NK, NKT, CD8^+^T, CD4^+^T cells and regulatory T cells (Tregs), as well as Ly6Chi inflammatory monocytes and dendritic cells. The lack of miR-155 led to increased parasite burden and decreased survival of infected mice in contrast to WT mice. Innate and adaptive immune responses were reduced in the absence of miR-155, along with decreased proinflammatory mediators, Th-1-associated and Th-2-associated cytokines and accumulation of lymphocyte subpopulations. Also, CD8^+^ T cell exhaustion was also worsened in the absence of miR-155 via targeting of SHIP-1 and SOCS1, showing as up-regulated recruitment of Tregs and expression of PD-1, and down-regulated expression of IFN-γ and TNF-α in CD8^+^ T cells. Our results show that miR-155 is a critical immune regulator for the control of *T. gondii* infection, suggesting that miR-155 can be explored as a potential molecular target for boosting immunity against *T. gondii*.

## Abstract (fr)

*Toxoplasma gondii* est bien connu pour infecter presque toutes les espèces aviaires et mammifères, y compris les humains, avec une distribution mondiale. Ce parasite protozoaire peut provoquer une toxoplasmose grave, présentant un risque pour la santé publique. Le rôle des microARN dans la pathogenèse de *T. gondii* n’a pas été bien décrit. Le but de la présente étude était d’étudier le rôle du microARN-155 (miR-155) dans la médiation des réponses immunitaires innées et adaptatives lors d’une infection à *T. gondii* dans des modèles de souris. La survie et la charge parasitaire chez les souris miR-155^−/−^ et de type sauvage C57BL6 infectées par *T. gondii* ont été comparées. Dans ces deux modèles de souris, des tests ELISA ont été utilisés pour l’analyse des cytokines associées à Th1, Th2 et Th17, et la cytométrie en flux a été utilisée pour l’analyse des sous-populations de cellules NK, NKT, CD8^+^T, CD4^+^T et les cellules T régulatrices (Tregs), ainsi que les monocytes inflammatoires Ly6Chi et les cellules dendritiques. L’absence de miR-155 a entraîné une augmentation de la charge parasitaire et une diminution de la survie des souris infectées contrairement aux souris de type sauvage. Les réponses immunitaires innées et adaptatives ont été réduites en l’absence de miR-155, ainsi qu’une diminution des médiateurs pro-inflammatoires, des cytokines associées à Th-1 et Th-2 et à une accumulation de sous-populations de lymphocytes. En outre, l’épuisement des lymphocytes T CD8^+^ s’est également aggravé en l’absence de miR-155 via le ciblage de SHIP-1 et SOCS1, se manifestant par un recrutement régulé à la hausse des Tregs et l’expression de PD-1, et une expression régulée à la baisse de l’IFN-γ et TNF-α dans les cellules T CD8^+^. Nos résultats montrent que miR-155 est un régulateur immunitaire essentiel pour le contrôle de l’infection à *T. gondii*, suggérant que miR-155 peut être exploré comme cible moléculaire potentielle pour renforcer l’immunité contre *T. gondii*.

## Introduction

As the causative agent of toxoplasmosis, *Toxoplasma gondii* can infect all warm-blooded animals, affecting approximately 30% of the world’s population [12, 25]. Primary infection in immune-potent individuals is usually asymptomatic or presents as a mild, flu-like illness, concomitant with parasite conversion to dormant bradyzoites within a tissue cyst [20]. However, severe toxoplasmosis in immunocompromised individuals can develop following reactivation of bradyzoites into disseminating tachyzoites, leading to toxoplasmic encephalitis (TE), eye disease, neurological problems and even death [12, 20]. Congenital infection can occur in newborns resulting from the infection of pregnant women, and is associated with dysplasia, hydrocephaly and chorioretinitis [11]. *Toxoplasma gondii* infection can also cause considerable economic losses to the stock-raising industry [12]. Although acute *T. gondii* infections can be controlled by medications, there are still no effective drugs that can be used to completely eliminate chronic infection, and more effective therapeutic agents are needed for this organism [9]. Additionally, no approved clinical vaccines are available for human infections [32]. Determining the immune response to *T. gondii* is vital for the design of effective vaccines and also to find drug targets.

It is well documented that the immune response induced by *T. gondii* infection is complex, including innate and adaptive immune response, which involves various immune cells, such as CD4^+^ and CD8^+^ T cells, natural killer (NK) cells, dendritic cells (DCs), macrophages and neutrophils [22, 36]. CD8^+^ T cells and their responses are essential for the control of infection by acting synergistically with CD4^+^ T cells [10]. Primarily, T helper 1 (Th-1) cell-mediated protective immunity to *T. gondii* drives the production of high levels of interleukin-12 (IL-12) and interferon-γ (IFN-γ) followed by the migration of innate immune cells (e.g., DCs, macrophages and neutrophils) to the site of infection, which are indispensable for host resistance against *T. gondii*, and limiting the parasite’s proliferation and the progression of infection via multiple intracellular mechanisms with the production of various antiparasitic factors [13, 23]. The expansion of Th-2 cells and anti-inflammatory cytokine production of IL-4, IL-10, IL-13, IL-27, and transforming growth factor-β (TGF-β) are responsible for controlling the effects of excessive immune activation and preventing immunologic pathology [23]. Collectively, these innate and adaptive immune responses, mainly Th-1 and Th-2-associated cytokines contribute to protective immunity against *T. gondii* infection.

MicroRNAs (miRNAs), as class of non-coding RNAs, are involved in gene regulation at both transcriptional and post-transcriptional levels [2]. Through several mechanisms, including translational repression and messenger RNA (mRNA) degradation, miRNAs have been shown to mediate and regulate some physiological processes, and also to be related to human disease, such as carcinogenesis, and even extended to the immune system [18, 19]. It has been revealed that miRNAs can regulate immune system biology, including both lymphocyte development and function and host immune responses, and also they have been shown to be involved in inflammation associated with CD4^+^ T cell differentiation and CD8^+^ T cell responses [19]. In particularly, miRNA-155 is a well-characterized miRNA known to be a key regulator of monocytes, macrophages, T-cells and B-cells by interference in the expression of pro-inflammatory and anti-inflammatory cytokines [1, 31]. miRNA-155 has a regulatory role in visceral leishmaniasis by up-regulating both Th-1 and Th-2 immune responses, which are together to be contributed to the control of the infection [30]. miRNA-155 has been demonstrated to be an important immune regulatory molecule for the control of *Trypanosoma cruzi* infection [16]. Likewise, miR-155 is known to play a critical role in maintaining the survival of Mtb-infected macrophages and the function of Mtb-specific T-cells during *Mycobacterium* infection [21].

Despite the fact that miR-155 is elevated in chronic *T. gondii* infection [34], its specific role in regulating innate and adaptive immune responses against this infection has not been elucidated. To determine whether miRNA-155 influences the progression of *T. gondii* infection and immune responses in mice models, we investigated the role of miRNA-155 during immunity to *T. gondii* infection in miR-155 gene-deficient mice and those of their age- and sex-matched wild-type (WT) counterparts. Our results show that miR-155 is required for control of *T. gondii* infection via mediating a wide spectrum of immune compartments.

## Materials and methods

### Ethics

All mice were maintained and bred in strict accordance with the Animal Ethics Procedures and Guidelines of the People’s Republic of China. Animal experiments were approved by the ethics committee of Ningbo University (permission: SYXK(ZHE)2019-0005).

### Mice and parasites

Age-matched 6–8 week old female miR-155KO C57BL/6 and WT C57BL/6 mice were purchased from Beijing Vital River Laboratory Animal Technology Co., China.

The PRU strain (Type II) of *T. gondii* was used for the *in vivo* challenge of mice, which was propagated and harvested as described in studies [35]. The tachyzoite forming of the PRU strain (Type II) of *T. gondii* were also used for preparation of soluble tachyzoite antigens (TLA), as previously described [33, 35].

### Cytokine ELISA

According to previously described methods [33, 35], splenocytes were prepared by pushing the spleens through a wire mesh, and then purified by removing red blood cells using RBC erythrocyte lysis buffer, and then re-suspended in DMEM medium supplemented with 10% FCS, 1% penicillin – streptomycin, and 1% HEPES. The test specimen was then plated at a concentration of 5 × 10^6^ cells/mL and stimulated for 72 h with 15 μg/mL TLA. Cell-free supernatants were collected and assayed for IL-2 and IL-4 at 24 h, for IL-22 activity at 48 h, for IL-13, IL-17A, IL-17F activity at 72 h, and for IFN-γ activity at 96 h, using commercial ELISA kits according to the manufacturer’s instructions (Biolegend, USA).

### Isolation of mouse spleen lymphocytes

After reaching the experimental detection conditions, the mice were sacrificed and spleens were taken out. Red blood cell lysate was used to lyse red blood cells, and 40% and 70% percoll solutions were prepared with percoll stock solution, and lymphocytes were separated by density gradient centrifugation.

### Flow cytometry

Single-cell suspensions were prepared according to the method mentioned above. Followed by blocking of FC receptors with addition of normal mouse serum, cells were stained with the following antibodies: CD3 (BV421, clone: 145-2C11, BD Biosciences), CD4 (BV510, clone: RM4-5, BD Biosciences), CD8 (PE, clone: 53-6.7, BD Biosciences), NK1.1 (PE-CY7, clone: PK136, BD Biosciences), CD11b (BV605, clone: M1/70, BD Biosciences), Ly6C (APC, clone: 560595, BD Biosciences). For intracellular cytokine staining (ICS) assays, prior to intracellular stained with IFN- and AF647 (clone: XMG1.2, BD Biosciences) for 30 min at 4° C, the cells were surface-stained CD8-PE (BD Biosciences), fixed and permeabilized with Cytofix/Cytoperm solution (BD Biosciences) for 20 min in dark, and data were then acquired by performing these cells on a Beckman CytoFLEX S and data were further analyzed by FlowJo software (Tree Star, Inc., Ashland, OR, USA).

### qRT-PCR

The expression of IL-1α, IL-1 β, IL-6, SHIP-1 and SOCS1 were analysed by qRT-PCR. Total RNA was isolated from three purified splenocytes of mice in each group using Trizol reagent (Invitrogen, USA), as per the manufacturer’s instructions. RNAs were dissolved in RNase-free ddH_2_O (TaKaRa, China) and cDNA was synthesized using a GoScript™ Reverse Transcription System (Promega, Madison, WI, USA) and used as a template for quantitative real-time polymerase chain reaction (qRT-PCR). qRT-PCR was performed using a Light Cycler 480 SYBR Green I Master (Roche, Switzerland). The primers used for amplification are listed in Table 1. qRT-PCR analysis was performed on the Light Cycler 480 (Roche, Switzerland) and data were calculated using the comparative cycle threshold (CT) method (2^−ΔCT^).

**Table 1:** Primer sequences.

Gene	Sequence (5′→3′)
SHIP-1	Sense: 5′–TCTTGCAACAGAGAACCCCC–3′
	Anti-sense: 5′–TCCTGGATGGCTTTCAGGTG–3′
SOCS1	Sense: 5′–TCTTGCAACAGAGAACCCCC–3′
	Anti-sense: 5′–TCCTGGATGGCTTTCAGGTG–3′

### Challenge and parasite burdens

A total of five mice in each group were challenged with 10 PRU tissue cysts of *T. gondii* PRU strain orally, and the survival periods were recorded daily until all mice were dead. In parallel, mean brain cyst loadings were counted at 14 days after the challenge, as described in our previous studies [33, 35]. All samples were counted in triplicate. The parasite reduction rate of brain cysts is relative to that of the control.

### Statistical analysis

Statistical analysis was conducted using GraphPad Prism 8. Student’s unpaired *t* test was used to determine statistical significance of differences among the groups. *p*-values < 0.05 were considered statistically significant.

## Results

### miR-155 deficiency enhanced the susceptibility to *T. gondii*

**Figure 1:**
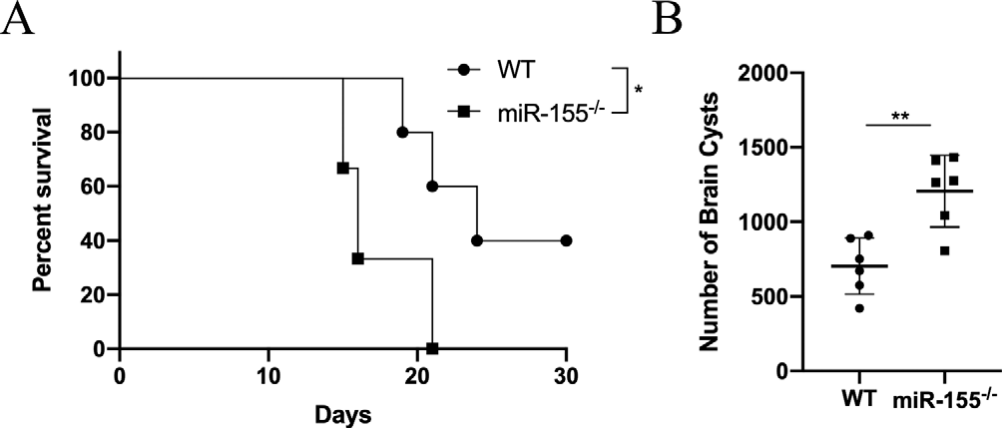
miR-155 deficiency enhanced cyst burden and reduced survival time in mice models. (A) Survival curves of mice after challenge of cysts of the PRU strain. miR-155^−/−^ mice had 0% survival at day 17. (B) Cyst loads were counted from whole brain homogenates of mice 4 weeks after challenge. The bars represented the mean cyst burden per mouse brain after challenge orally with a dose of 10 cysts of the PRU strain. Data are mean ± SD (representative of three experiments). **p < 0.01; *p < 0.05, compared with the WT groups.

miR-155 was recently found to contribute to resistance to the experimental parasitic diseases [16, 30]. To test whether the lack of miR-155 would potentiate chronic *T. gondii* in mice models, miR-155^−/−^ mice were orally inoculated with 10 PRU tissue cysts. All of the miR-155^−/−^ infected mice died after 21 days of infection, but WT mice infected with *T. gondii* showed a prolonged survival time (Fig. 1A). To further confirm the *T. gondii* infection in WT and miR-155^−/−^ mice, the mean brain cyst loadings were counted. *Toxoplasma gondii*-infected miR-155^−/−^ mice showed more brain cysts (Fig. 1B). These data demonstrate that the absence of miR-155 enhances susceptibility to *T. gondii* in mice models.

### miR-155 deficiency impairs both Th-1 and Th-2 immune responses in the spleens of *T. gondii*-infected mice

As miR-155 has been shown to be critical for regulating T-cell responses as well as inflammatory responses and cytokine signals, which have well-documented roles in the control of intracellular pathogens, including *T. cruzi* and *Leishmania donovani* [16, 30], we further analysed the production of cytokines Th-1-associated IFN-γ, IL-2, Th-2-associated IL-4 and IL-13, Th-17-associated IL-17A and IL-17F by splenic cells harvested from WT and miR-155KO (miR-155^−/−^) mice after infection with *T. gondii* PRU cysts.

**Figure 2:**
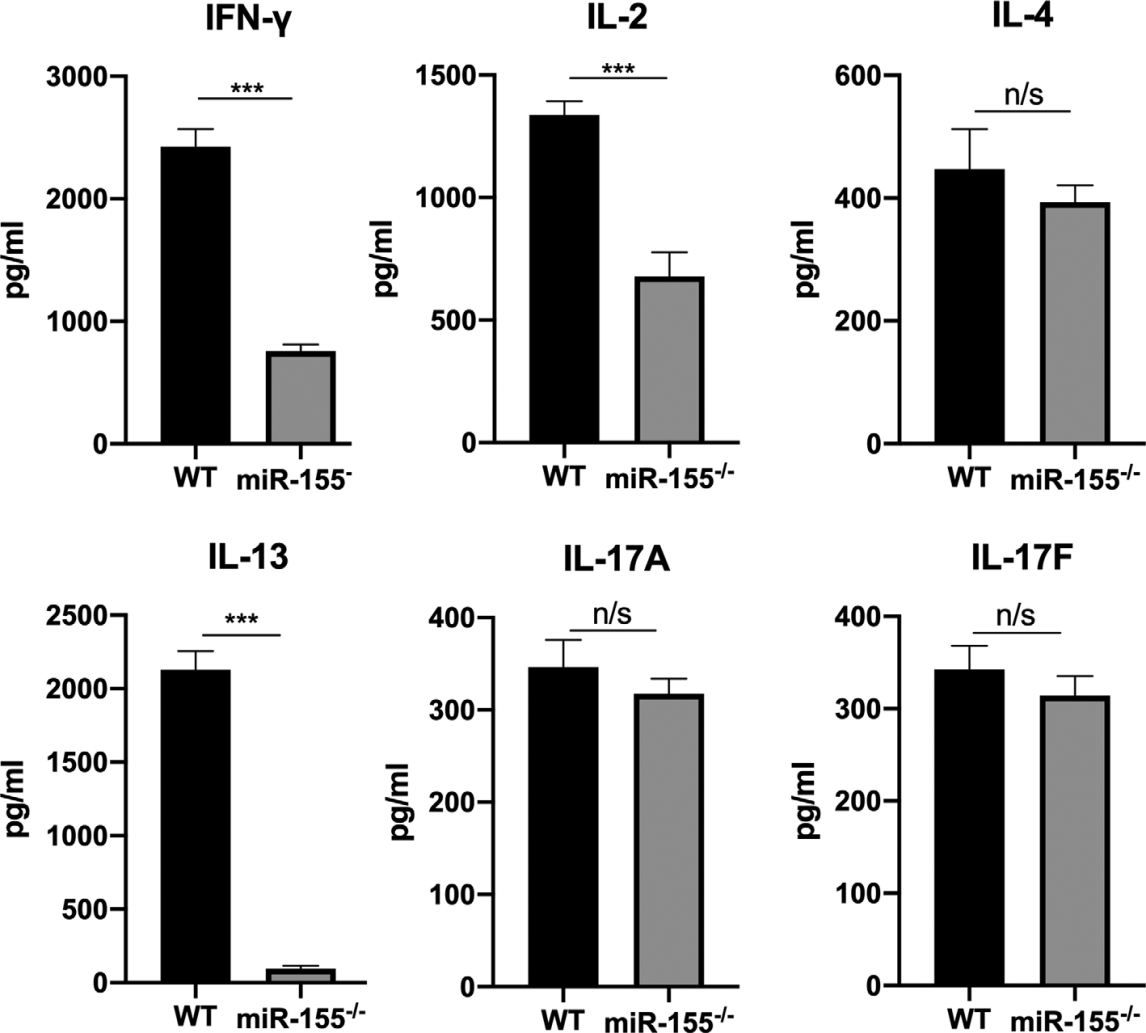
Evaluation of Th-1\Th-2\Th-17 immune responses in WT and miR-155^−/−^ mice. The levels of IFN-γ, IL-2, IL-13, IL-4, IL-17A and IL-17F were measured from splenic lymphocytes of WT- and miR-155KO-infected mice. Significant decreases in IFN-γ, IL-2, IL-13 were observed in miR-155^−/−^ mice. Data are shown from one representative experiment of three independent experiments and presented as a mean ± SD. ***p < 0.001; N/S = not significant.

After ELISA analysis of TLA-stimulated spleen cells supernatants, it was shown that the production of IFN-γ and IL-2 was significantly reduced in *T. gondii*-infected miR-155^−/−^ mice compared to WT controls (Fig. 2). Likewise, IL-13 production by splenic cells from *T. gondii*-infected miR-155^−/−^ mice was dramatically decreased in contrast to WT controls (Fig. 2). However, there were no significant changes in the levels of IL-4, IL-17A and IL-17F in these *T. gondii*-infected miR-155^−/−^ mice and WT controls (Fig. 2). Taken together, these data suggest that this *T. gondii* infection in miR-155^−/−^ is due to lower protective cytokine production.

### Lack of miR-155 contributes to impaired recruitment of CD4^+^ T, CD8^+^ T, NK and NK-T cells in the spleens of *T. gondii*-infected mice

**Figure 3:**
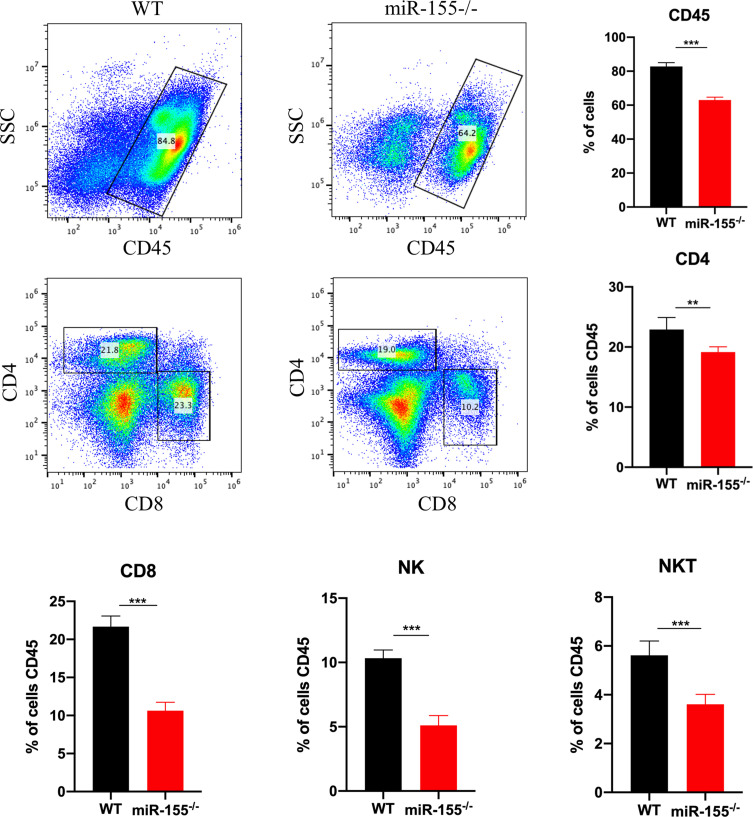
miR-155 deficiency impairs the recruitment of CD4^+^ T cells, CD8^+^ T cells, NK and NKT cell. Spleens were collected from each mouse in the WT and miR-155^−/−^ groups. The splenocytes labeled with cell-specific fluorescent labeled antibodies were analysed by flow cytometry. Results were calculated and expressed as a mean ± SD of three independent experiments. ***p < 0.001; **p < 0.01.

NK cells, NK-T, and T cells are usually recruited to kill *T. gondii* by hosts through the production of IFN-γ and a perforin-independent mechanism [13, 23]. To investigate whether miR-155 deficiency has an effect in recruitment of these immune cells, the splenic cell populations of CD4^+^ T cells, CD8^+^ T cells, NK cells and NK-T cells were analysed from *T. gondii*-infected miR-155^−/−^ and WT mice by flow cytometry. We found that these splenic cell populations of *T. gondii*-infected miR-155^−/−^ mice were significantly decreased in contrast to WT counterparts (Fig. 3). These results suggest that miR-155 plays a role in mediating recruitment of NK, NK-T cells, CD4^+^ T cells and CD8^+^ T cells, which control *T. gondii* infection, while these decreased recruited cells in miR-155^−/−^ mice could not activate protective immunity effectively, leading to subsequent aggravated parasitic infection and even death.

### miR-155 deficiency exacerbated CD8^+^ T cell exhaustion

**Figure 4:**
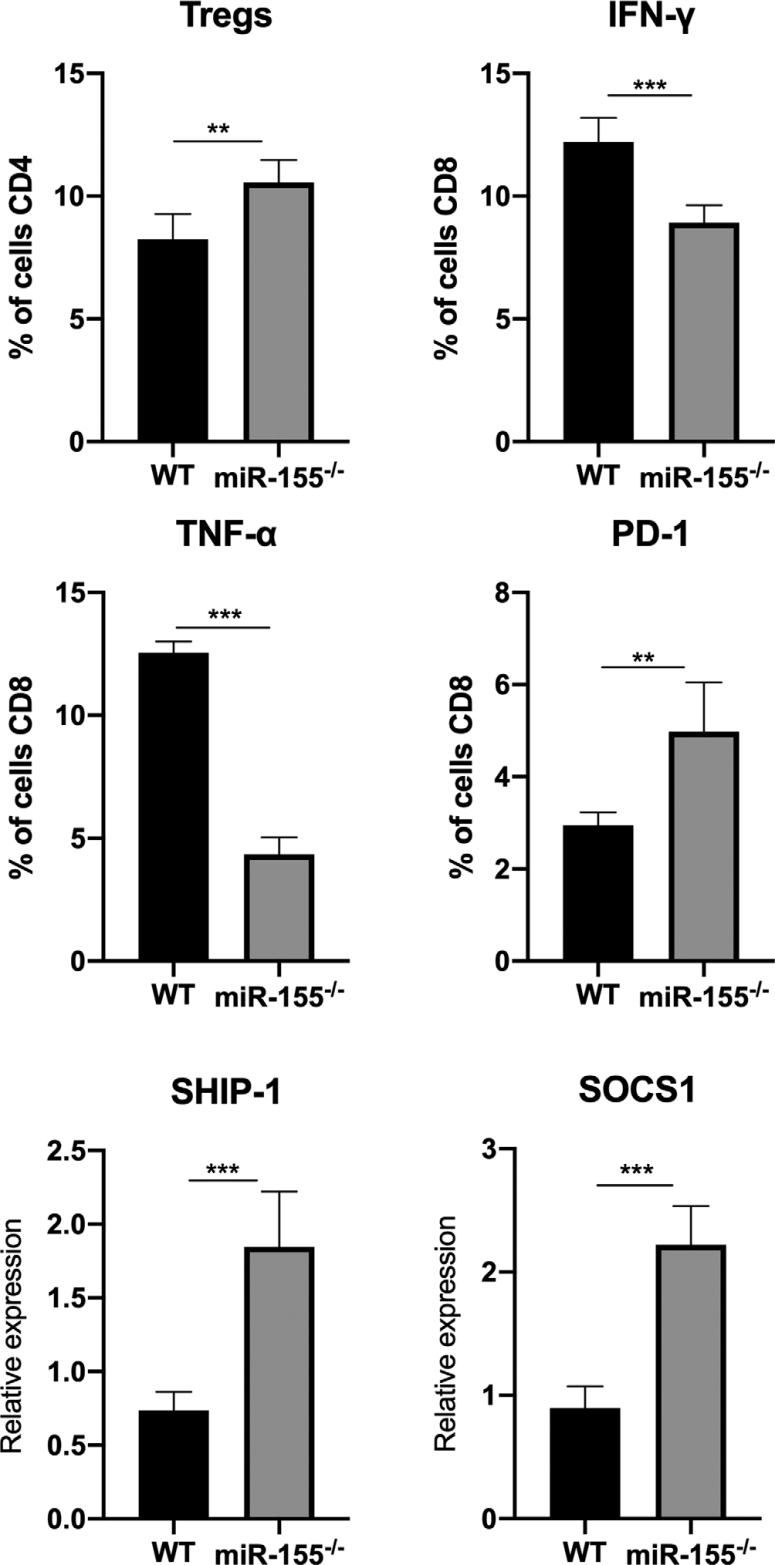
miR-155 deficiency exacerbated CD8^+^ T cell exhaustion by targeting to SHIP-1 and SOCS1. Spleens were collected from each mouse in the WT and miR-155^−/−^ groups. The percentages of Tregs, IFN-γ CD8^+^ T, TNF-α CD8^+^ T and PD1 CD8^+^ T cells were analysed by flow cytometric analysis. The expressions of SHIP-1 and SOCS1 were estimated by qRT-PCR. Data are shown from one representative experiment of three independent experiments and presented as a mean ± SD. ***p < 0.001; **p < 0.01.

miR-155 has recently been found to restrain CD8^+^ T cell functional exhaustion in chronic virus infection and tumors, by targeting several inhibitors of cytokine signaling, including SOCS1 and SHIP1 [8, 24]. Thus, to better understand the role of miR-155 on CD8^+^ T cell exhaustion during chronic infection with *T. gondii*, we analysed the expression of IFN-γ, TFN-α and PD-1 in CD8^+^ T cells and the number of regulatory cells (Tregs) by flow cytometry (Fig. 4). As we expected, in miR-155KO mice, there were significantly decreased numbers of IFN-γ CD8^+^ and TNF-α CD8^+^ T cells in contrast to WT mice, suggesting decreased effector T cells in miR-155KO mice (Fig. 4). Similarly, the number of Tregs were also up-regulated in *T. gondii*-infected miR-155KO mice, suggesting enhanced immune-inhibitory effects followed by the deficient expression of miR-155.

It is known that PD-1 expression by the myeloid cell population plays an important role in immune regulation in various infectious diseases as well as in several cancer models [15, 17, 29]. Since PD-1 has been implicated in suppressing T cell response in *T. gondii* [4], flow cytometric analysis of the expression PD-1 in CD8^+^ T cell revealed that *T. gondii-*infected miR-155KO mice up-regulated the expression PD-1 in CD8^+^ T cell in contrast to *T. gondii-*infected WT mice. Furthermore, miR-155KO mice showed increased expression of both SHIP-1 and SOCS1 by qRT-PCR (Fig. 4), which are known to be direct targets of miR-155 [8, 24]. Together, these findings indicate that miR-155 deficiency contribute to increased T cell exhaustion by suppressing T cell responses by targeting SHIP-1 and SOCS1.

### miR-155 deficiency decreased accumulation of splenic inflammatory monocytes and DCs and expression of pro-inflammatory mediators

**Figure 5:**
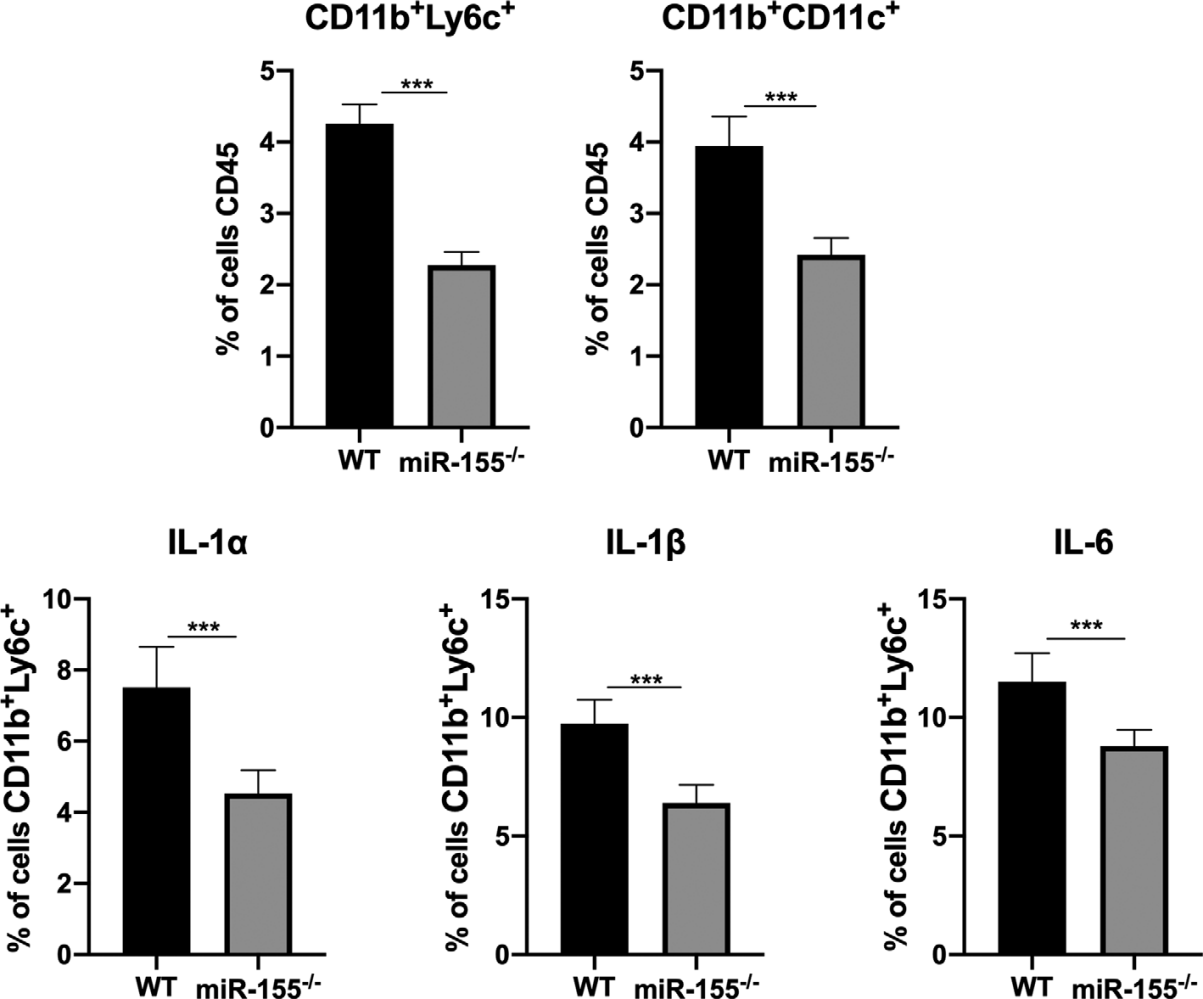
Decreased accumulation of splenic inflammatory monocytes and DCs and expression of pro-inflammatory mediators in miR-155KO-infected mice. Flow cytometric analysis of splenic inflammatory monocytes and DCs of parasite infected-WT and miR-155^−/−^ mice. The expression of pro-inflammatory mediators was analysed by qRT-PCR. Data are presented as a mean ± SD of three independent experiments. The asterisks indicate the statistical significance of difference between WT and miR-155^−/−^ mice. ***p < 0.001; **p < 0.01.

Recent studies have established the critical role of CD11b^+^Ly6C^+^ cells and DCs in *T. gondii* infection [5, 26]. Flow cytometric analysis of phagocyte populations has revealed that *T. gondii*-infected miR-155^−/−^ mice contained significantly lower numbers of CD11b^+^Ly6C^+^ cells and DCs than their WT counterparts (Fig. 5). We further determined whether decreased numbers of CD11b^+^Ly6C^+^ cells and DCs in miR-155-KO mice consequently impaired the expression of pro-inflammatory mediators by flow cytometric analysis. Consistent with decreased numbers of CD11b^+^Ly6C^+^ cells and DCs, the expression of IL-1α, IL-1β and IL-6 was significantly reduced in contrast to their *T. gondii*-infected WT counterparts (Fig. 5). These data suggest that miR-155 deficiency leads to decreased accumulation of DCs and Ly6C^+^ inflammatory monocytes, in combination with reduced production of pro-inflammatory mediators that could contribute to high parasitic burdens in miR-155KO mice.

## Discussion

It is well known that both IFN-γ and IL-4-associated signaling pathways are desired immunological responses to defend against *T. gondii* infection [22]. In particular, releasing of Th-1 type cytokines, IL-12, IL-2 and IFN-γ are essential for the development of T cell immunity against *T. gondii* infection [13]. Likewise, T helper type 2 (Th-2) cell-associated cytokines, such as IL-4, IL-10 and IL-13, also play an important role in coordinating the immune response by dampening systemic Th-1 type cytokine production and thus prevent lethal immunopathology [10]. Th-17-associated immune responses are also known to provide significant protective immunity against *T. gondii* infection [14]. Although miR-155 has been shown to be able to regulate the development and activity of Th-1, Th-12, and CD8^+^ T cells, its specific role in immunity against *T. gondii* infection has yet to be well studied. In our study, we established that miR-155 contributes to host immunity against *T. gondii* infection through the regulation of Th-1 and Th-2 immune responses, but it showed no any effect in Th-17-associated immune responses.

Some recent studies have shown the important role of microRNAs during infection [6, 8, 24]. In our study, it was found that a lack of miR-155 led to a significant increase in parasitic brain cysts and decreases in survival times. Since miR-155 has been shown to be the key regulator of IFN-γ production via the targeting of SOCS1 [3], it is not surprising that this regulative effect should be ascribed to the inability of miR-155^−/−^ mice to mount an efficient Th-1 immune response, with the reduced production of IFN-γ and IL-2. Additionally, we observed a decrease in Th2 immune responses in miR-155^−/−^ mice, which is similar to a previous study *in vitro* in *L. donovani*-infected miR-155^−/−^ mice [16], indicating that miR-155 is potentially beneficial for the generation of optimal Th-2 immune responses against *T. gondii* infection. These data demonstrate that miR-155 plays a significant role in the regulation of both Th-1 and Th-2 immune responses during *T. gondii* infection.

miR-155 regulates the activation of several immune subpopulations including CD8^+^T cells, as well as NK and NKT cells [27, 28], which are critical for protective immunity against *T. gondii* infection [14]. Previous studies have found that miR-155 is important in the control of *Leishmania donovani* and *T. cruzi* infection by mediating the regulation of T-cell proliferation and thus the activation of CD8^+^ T cells, NK and NKT cells [16, 30]. In support of these findings, our data indicate that decreased recruitment of CD8^+^ T cells, NK and NKT cells resulting from the absence of miR-155 expression, can lead to the lack of control of parasite infection in the miR-155^−/−^ mice.

As the first line cell types in the initial stages of infection, inflammatory monocytes, especially Ly6C^+^ monocytes are considered to be necessary to govern the control of chronic infection with *T. gondii* in mice [5], through the inductive production of proinflammatory mediators, such as IL-1α, IL-1β, IL-6, inducible NO synthase, TNF, and reactive oxygen intermediate. Additionally, DCs are essential for the expansion and subsequent T cell priming and activation of Ag-specific CD8^+^ T cells during infection with *T. gondii* [26]. Our studies have shown decreased accumulation of Ly6C^+^ inflammatory monocytes and DCs in miR-155KO mice, associated with a significantly increased parasite load, together with the reduced expression of these proinflammatory mediators. However, it is contradictory that aberrant miR-155 expression has recently been shown to adversely affect Ly6C^+^ inflammatory monocytes migration in *L. donovani* and *T. cruzi*, which is due to a fact that inflammatory monocytes facilitate the growth of the parasites in the spleen in visceral leishmaniasis and trypanosomosis [16, 30]. These results demonstrate that miR-155 deficiency exacerbated *T. gondii* infection through the down-regulation of DCs and inflammatory monocyte infiltration.

Recent studies showed that chronic infection with *T. gondii* led to CD8^+^ T cell exhaustion, concomitant with decreased CD8^+^ T cell effector response, which is characterized as up-regulated expression of inhibitory receptor PD-1 on these CD8^+^ T cells [4]. miRNA-155 expression is essential for optimal CD8^+^ T cell responses toward chronic infection with LCMV, and cancer, involved in regulation of CD8^+^ T cell exhaustion [8, 24]. In this study, our analysis of the effects of miR-155 on cellular immune responses showed that the deficiency of miR-155 ablated CD8 T cell responses during chronic *T. gondii* infection, along with up-regulation of PD-1 and down-regulation of IFN-γ and TNF-α in CD8^+^ T cells. In the meanwhile, Tregs were also augmented in miR-155^−/−^ mice, which have been shown to act as immune-counter in cancer [7], indicating a possible immune-inhibitor effect in the infection with *T. gondii*, but its specific role needs further confirmation. As the direct target of miR-155, SHIP-1 and SOCS1 are indicated as negative regulators of IFN-γ production and T cell exhaustion [8, 24]. The higher SHIP-1 and SOCS1 levels in infected miR-155KO mice, together with the disrupted responses of CD8^+^ T cells suggest that T cell exhaustion during chronic *T. gondii* infection is mediated by miR-155-dependent mechanisms.

## Conclusions

We have demonstrated that the absence of miR-155 could enhance susceptibility to chronic *T. gondii* infection in mice models. It is clear that this effect of miR-155 is ascribed to its contribution to host immunity via the regulation of both Th-1 and Th-2 protective immune responses, as well as inflammatory monocyte and DC infiltration. It should be noted that miR-155 was essential to mediate T cell exhaustion in this model. Therefore, miR-155 can be exploited as a potential target for reinforcing host immunity against *T. gondii*.

## Funding information

Public Welfare Science and Technology Plan Project of Ningbo (202002N3153), Ningbo medical and health brand discipline (PPXK2018-06), Children’s Health and Disease Clinical Medicine Research Center of Ningbo (2019A21002).

## Conflict of interest

The authors declare that they have no conflict of interest.
